# Evaluation of bacteriophage efficacy in reducing the impact of single and mixed infections with *Escherichia coli* and infectious bronchitis in chickens

**DOI:** 10.1080/20008686.2019.1686822

**Published:** 2019-11-29

**Authors:** Maram M. Tawakol, Nehal M. Nabil, Ahmed Samy

**Affiliations:** Reference Laboratory for Veterinary Quality Control on Poultry Production, Animal Health Research Institute, Giza, Egypt

**Keywords:** Bacteriophage, *E. coli*, IBV, mixed infection

## Abstract

Infectious bronchitis virus (IBV) represents a major threat to poultry production worldwide particularly when complicated with bacterial infection. In the present study samples were collected from forty broiler farms with respiratory manifestations to characterize IBV and *E. coli*. Bacteriophages were isolated and enriched from sampled farms to study its efficacy to control single and mixed infections with *E. coli* and IBV *in vivo. *Twelve out of forty farms were positive for IBV. Phylogenetic analysis of partial spike protein revealed that all positive cases clustered within the GI-23 genotype. Eight out of forty farms were positive for *E. coli* serogroups O26, O78, O86, O114, O119, with O125 found on three farms. Bacteriophage treatment delayed the onset and reduced the severity of clinical signs, and prevented the mortality associated with single and mixed infection with IBV and *E. coli.* Furthermore, in mixed infections, bacteriophage treatment significantly reduced *E. coli* as well as IBV shedding. Groups treated with bacteriophages showed a significant reduction of *E. coli* shedding that gradually decreased over time, in contrast to higher and gradually increasing shedding without bacteriophage treatment. In conclusion, bacteriophage treatment significantly reduced the pathogenicity and shedding of IBVand *E. coli* in mixed infections.

## Introduction

Avian respiratory tract infections are associated with massive economic losses particularly under poor intensive rearing conditions in winter seasons. Several pathogens, adverse environmental conditions and poor managemental factors are involved [1–3]. Pathogens include avian influenza virus (AIV), Newcastle disease virus (NDV), infectious bronchitis virus (IBV), avian pneumovirus (APV), *Mycoplasma gallisepticum* (MG), and avian pathogenic *E. coli* (*APEC)*. Avian respiratory tract infections can be caused by single pathogens or in combination with each other in form of preceding, concurrent or secondary infections [3–5]. Change in the ecosystem of the respiratory microbiome [6], mechanical damage of the epithelium, loss of ciliated cells and the impairment of innate immune responses are a consequence of primary respiratory infections and associated with enhanced disease and economical losses during superinfections [7].

In Egypt, respiratory diseases outbreaks caused significant economic losses in commercial poultry production during recent years [8]. Laboratory investigations revealed that IBV represents the most common pathogen detected during these outbreaks [8–10]. IB is a highly contagious disease affecting the respiratory, renal and reproductive tract of chickens. The disease caused by single-stranded, enveloped RNA virus belongs to the genus of Gamma coronavirus [11]. The spike (S) glycoprotein represents the major structural and immunogenic domain, and plays a pivotal role in tissue tropism and carry the receptor-binding site [12]. The S glycoprotein is cleaved post transitionary into S1 (globular head) and S2 (stalk) domain [13]. The S1 domain possesses three hypervariable regions (HVR 1, 2 and 3) responsible for the high genetic and antigenic variability of IBV [14]. Despite massive vaccination strategies used in Egypt, IBV extensively circulates in all over Egypt in vaccinated and non-vaccinated chickens with continuous viral evolution [9,15,16]. Complications between *APEC* and IBV infections are widely observed under field conditions and are reproduced under experimental conditions resulting in enhanced severe colibacillosis [7] and enhanced pathogenicity post IBV infection.

Under field conditions, commercial broiler farms with inadequate biosecurity measures and with the presence of colibacillosis in early age bird are associated with more severe IBV infections in late age birds (>20 days old). Despite the wide spread use of antibiotics and vaccination against IBV, complications as a consequence of mixed infections of avian bacterial pathogens including APEC and IBV became a general phenomenon associated with significant economic losses.

In the present study samples were collected from broiler farms with birds with respiratory manifestations. All samples were tested for the presence of IBV and APEC. Recovered IBV isolates were genetically characterized by partial spike protein sequencing. Recovered APEC were characterized by serotyping and antibiotic profiling. Bacteriophages were isolated from water samples collected from the same farms and enriched against APEC O78. *In vivo* study was conducted to evaluate the efficacy of bacteriophages in reducing the pathogenicity of single and mixed infections with APEC and IBV.

## Material and methods

### Sampling and sample preparation

One hundred recently succumbed chickens suspected of being IBV infected were collected from forty broiler farms. The study was conducted between late 2017 and early 2018 and samples collected in northern Egypt (Dakahila and Damietta governorates). Lung, trachea, kidney, and liver samples were aseptically collected. On the same day of sampling, half of the organs were cultured for bacteriological investigation in buffered peptone water at 37ºC for 24 h. For IBV screening, lung and trachea samples were homogenized with an equal mass of phosphate buffer saline and then centrifuged at 7,000 × *g* for 5 min. Supernatant was collected and stored at −80ºC until further investigation.

### IBV and E.coli detection and characterization

Viral RNA was extracted using the QIAamp viral RNA mini kit (QIAGEN) in accordance with the manufacturer instructions. One-step RT-PCR was performed for IBV screening as previously described [17]. Genotyping with forward and reverse primers for amplification of HVR1, amplicon purification and sequencing was performed as previously described [17]. Obtained sequences in this study aligned with sequences represent all infectious bronchitis virus genotypes proposed by [18]. All samples used in the phylogenetic analysis were downloaded from GenBank (http://www.ncbi.nlm.nih.gov). The sequences were aligned using multiple alignment MAFFT version 7 (https://mafft.cbrc.jp/alignment/server/). The tree was constructed with the MEGA 6 software [19] using the nucleotide substitution of the Hasegawa-Kishino-Yano model with the gamma-distributed rate (with four rate categories) with bootstrap value based on 1000 replicates [20]. Then, the tree was viewed and edited using the FigTree v1.4.2 software (http://tree.bio.ed.ac.uk/software/figtree/).

For the bacteriological investigation, *E.coli* isolation and confirmation was performed in accordance with [21]. Confirmed, positive E.*coli* strains were subjected to serotyping using all available O (O1 to O181) antisera in accordance with [22], cross-reacting antigens were used to ensure the removal of cross-reactivity. All confirmed strains were tested against antibiotics commonly used in Egyptian farms by disc diffusion, with testing procedures and interpretations of the results performed in accordance with reference laboratory protocols. One of the field isolates *E.coli* (serotype O78) was tested for purity using API 20E (bioMérieux, Inc.) and counted in colony-forming units (CFU).

### Bacteriophage isolation, enrichment, and titration

Over the surveillance period, sewage water samples were collected from poultry farms and used for the isolation of bacteriophages specific to APEC O78. Samples were centrifuged at 10,000 rpm for 10 min and then filtered through a 0.22 μm filter (Millipore). Bacteriophage purification and enrichment was performed in accordance with [23]. Briefly, filtered samples were mixed with Luria Bertani (LB) broth (Sigma), early-log grown APEC O78 added and samples incubated overnight at 37ºC with shaking set to 120 rpm/min. Then, samples were centrifuged at 10,000 rpm for 10 min and filtered through a 0.22 μm filter. The presence of bacteriophages was initially tested by the spot test method based on the double layer plaque technique in accordance with [24]. For this, 100 µl of APEC O78 were cultured on LB agar for 8 h, then 10 µl of the prepared bacteriophage suspension spotted onto it, and plates incubated at 37ºC overnight. The appearance of a clear zone in the plate indicated the presence of the lytic phage. Plaque assay was used for the titration of bacteriophages as previously described [25].

### *In vivo* evaluation of bacteriophage efficacy in the reduction of E.coli and/or IB pathogenicity

#### Ethics statement

Animal experimental designs and procedures were reviewed and approved by *RLQP* in accordance with guidelines of the ministry of agriculture and land reclamation and ministry of environment of Egypt.

#### Virus and bacteria

Pure *E.coli* O78 isolate recovered in the present study were titrated by colony-forming units (CFU). Birds were challenged with 10^8^ CFU diluted in 100 µl sterile PBS. The IBV strain IBV-EG/1212B-2012 (accession number: JQ839287) belonging to Egy/Var II [15] was used for the intratracheal challenge with 10^6^ EID_50_ in 100 µl per bird. Previously described primers and probes were used to create a standard curve for absolute quantification of IBV shedding [26].

#### Experimental design

**Figure 1.**
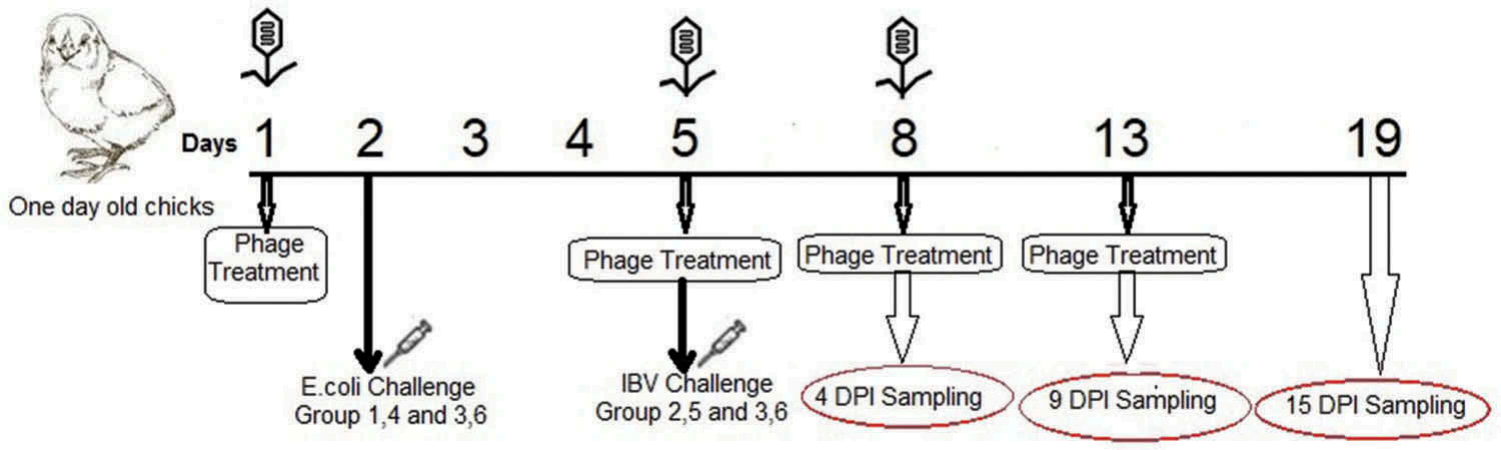
Schematic outline of the experimental design

A total of 70 healthy one-day old chicks were purchased from a local poultry hatchery and housed in isolators with *ad libitum* access to feed and water. One day later, chicks were monitored for the presence of IBV or APEC O78 as previously described and then randomly divided into seven groups. Groups 1, 2, and 3 were treated with bacteriophages by intratracheal inoculation with 10^8^ PFU at 1, 5, 8 and 13 days; whilst groups 4, 5, 6 and 7 were not treated with bacteriophages. Groups 1 and 4 were challenged intratracheally at day 2 with 10^8^ CFU APEC O78. Groups 2 and 5 were challenged intratracheally with 10^6^ EID_50_ of IBV. Groups 3 and 6 were infected with both pathogens in a 3 days interval. Group 7 was kept as a negative control and received PBS. The experimental design is shown in Figure 1. Three birds per time point were humanely culled at 4, 9 and 15 days post-infection (dpi). The carcasses were weighed and dissected and trachea and lung samples collected aseptically for the detection and titration of APEC O78, bacteriophages and IBV as previously described.

#### Clinical signs scoring and sampling

All birds were observed twice daily and clinical signs recorded in accordance with [27]. Briefly, no signs were recorded as 0, mild signs included mild gasping, coughing or depression and were recorded as 1, and severe gasping, coughing or depression with ruffled feathers was considered as severe signs and recorded as 2. Birds with severe signs unable to move were recorded as 3 and humanely culled and samples collected. Birds which unexpectedly succumbed to disease were also recorded as 3 and samples collected.

#### Statistical analysis

Statistical significances of viral and bacterial shedding between different groups were evaluated using Student’s t-test in Microsoft Excel based on the mean of shedding quantities in three tested birds sampled from each group at each time point.

## Results

### History, clinical findings, and prevalence of APEC and IBV in the examined flocks

Forty broiler flocks, aged between 30 and 40 days and suffering from respiratory affections, from the Dakahlia and Damietta governorate were examined. Unwell birds showed respiratory manifestations visible as nasal discharge, sneezing, gasping and swollen head. In all farms examined unwell birds were isolated and subjected to intense antibiotic treatments. However, clear differences in activity and feed intake remained. Screening of collected samples focused on the detection of IBV and *E.coli*.

Eight farms were positive for *E.coli* (20% of all farms examined), five farms from the Dakahlia and three farms from the Damietta governate. Serotyping of the isolated strains revealed the presence of the O26, O78, O86, O114, O119 serogroups, with the O125 serogroup detected on three farms. Disc diffusion tests showed the following resistance rates: 100% to penicillin, 75% to gentamycin and streptomycin, 62.5% to ciprofloxacin, 50% to cefotaxime and 25% to doxycycline. 75% of all isolated strains were multidrug-resistant (MDR).

**Figure 2.**
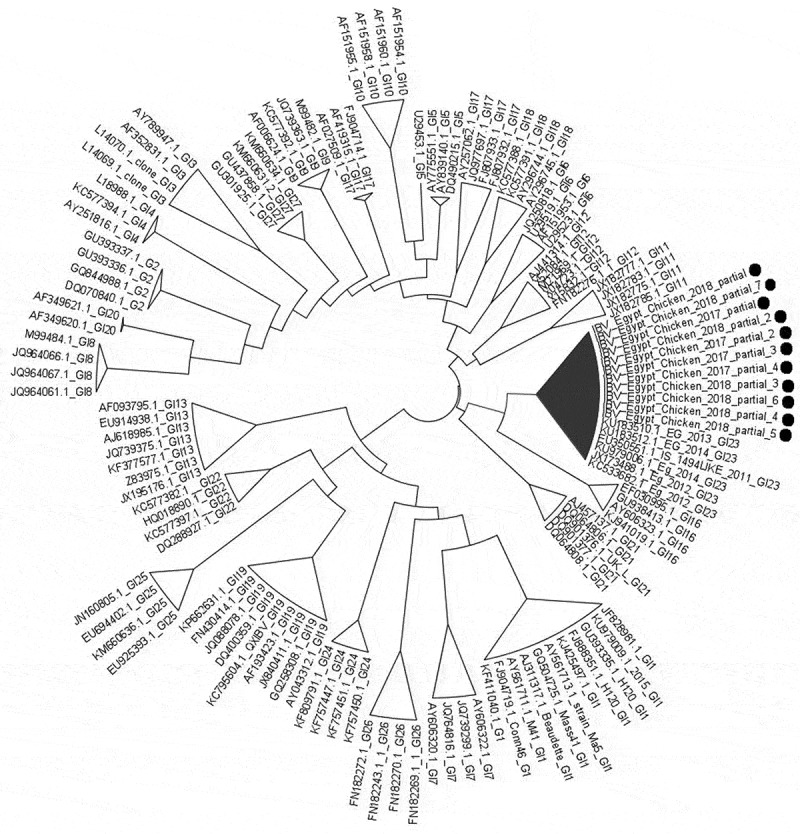
Phylogenetic analysis of IBV samples based on partial HVR1 sequences. Shown sequences represent all infectious bronchitis virus genotypes (GI) proposed by [18] with samples from the present study indicated by black circles. The tree was constructed using the nucleotide substitution of the Hasegawa-Kishino-Yano model with the gammadistributed rate (with four rate categories) with bootstrap value based on 1000 replicates

Twelve farms were positive for IBV (30% of all farms examined) determined by real-time PCR, with ten farms from the Dakahlia and two farms from the Damietta governate. Postmortem examination of the recently succumbed birds revealed the presence of caseous plug at the tracheal bifurcation, caseous trachitis, and fibrinous pericarditis, perihepatitis and air sacculitis. For virus isolation, specific pathogen free embryonated chicken eggs were inoculated. Total RNA was extracted from the allantoic fluid and the partial S1 gene of each isolate sequenced. Results obtained revealed that the tested samples clustered within the Egyptian variant group of GI-23 genotype subgroups (Figure 2). Nucleotide sequences tested in this study were designated as IBV_Egypt_Chicken_2017_partial, IBV_Egypt_Chicken _2018_ IBV_Egypt_Chicken_2017_partial_2, IBV_Egypt_Chicken_2017_partial_3, IBV_ Egypt_Chicken_2017_partial_4, IBV_Egypt_Chicken_2017_partial_5, IBV_ Egypt _Chicken_2018_partial_2, IBV_Egypt_Chicken_2018_partial_3, IBV_Egypt_ Chicken_2018_partial_4, IBV_Egypt_Chicken_2018_partial_5,IBV_Egypt_Chicken _2018_partial_6 and IBV_Egypt_Chicken_2018_partial_7, and were submitted to GenBank under accession number MK415385 and MK415386, MK882512, MK882513, MK882514, MK882515, MK882516, MK882517, MK882518, MK882519, MK882520 and MK882521 respectively.

### *In vivo* evaluation of bacteriophage treatment

**Figure 3.**
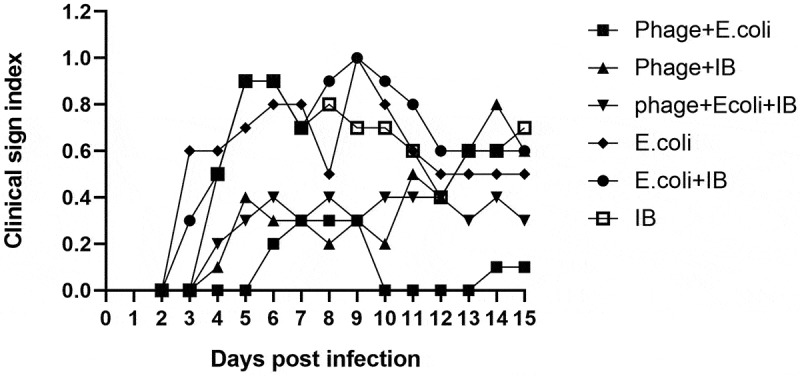
Clinical signs scoring in different groups. No signs were recorded as 0, mild signs included mild gasping, coughing or depression and were recorded as 1, and severe gasping, coughing or depression with ruffled feathers was considered as severe signs and recorded as 2. Birds with severe signs unable to move were recorded as 3 and humanely culled and samples collected. Birds which unexpectedly succumbed to disease were also recorded as 3 and samples collected. Each dot represents the mean of the clinical signs scoring of all birds in the group at each time point.

Clinical signs observed including gasping, coughing or depression started to appear from three days post-challenge with APEC or a mixed APEC and IBV infection. Bacteriophage treatment delayed the onset of the clinical signs to 6 days post-challenge (dpc) and in addition markedly reduced their severity in both groups (Figure 3). Regarding IBV infection, clinical signs were observed from four-days post-challenge, with bacteriophage treatment leading to a reduction of their severity, but not delaying their onset (Figure 3).

**Figure 4.**
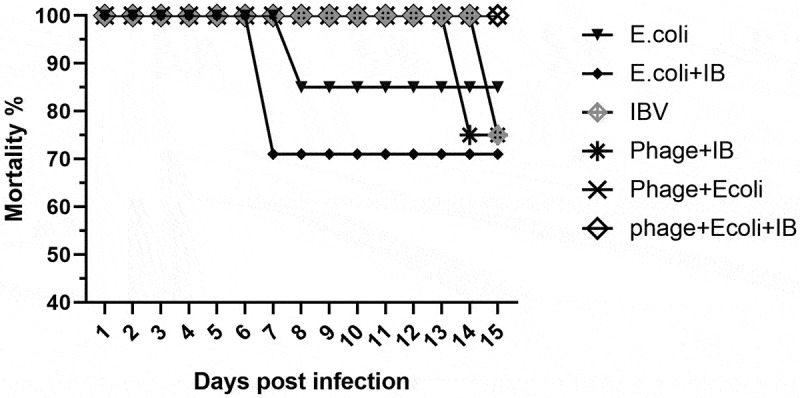
Average percentage of mortality in different groups (birds per group at each time point). The highest and earliest mortality was associated with mixed infections without bacteriophage treatment. In contrast, no deaths occurred in bacteriophage treated groups with mixed infection.

Bacteriophage treatment was not associated with mortality in single APEC or mixed APEC and IBV infected groups. In contrast, birds challenged with APEC alone and mixed APEC and IBV infection without bacteriophage treatment showed a 16% and 29% mortality rate at 8 and 7 days post-infection respectively (Figure 4). Bacteriophage treatment in combination with single IBV infection did not reduce the mortality of 26% (Figure 4).

**Figure 5.**
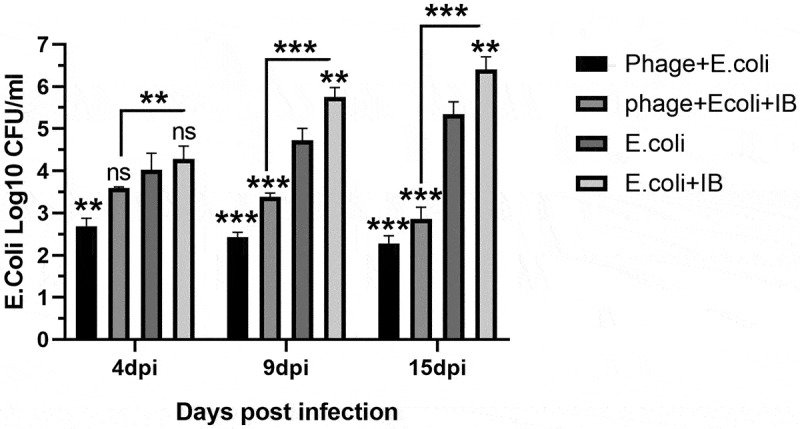
the E.coli bacterial counts. Samples were collected at 4, 9 and 15 dpc and are represented by the mean log10 CFU/ml of samples collected and tested from three birds. T-tests were performed. * p < 0.05, * * p < 0.01, ***p < 0.001 or ns for non-significant. The asterisks over columns represent a significant difference comparing to APEC the infected group. Asterisks over lines represent a significant difference between the mixed infection groups with and without bacteriophage treatments. Significantly higher counts with gradually increased bacterial shedding are observed in non-treated groups.

**Figure 6.**
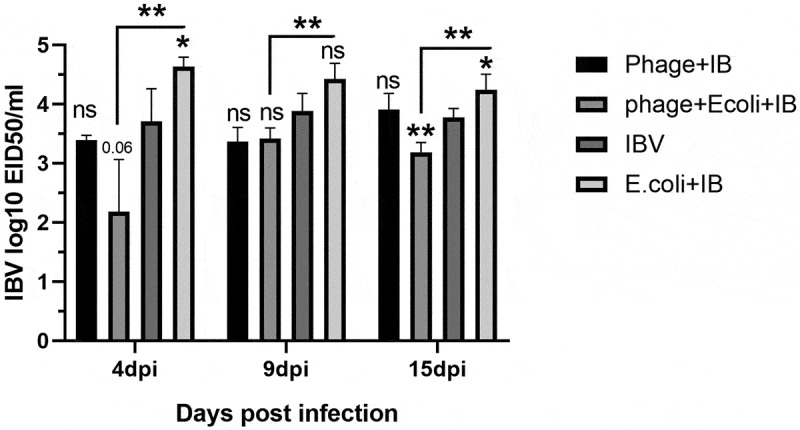
IBV titers. The IBV titers of samples collected at 4, 9 and 15 dpc are represented by the mean log10 EID50/ml of samples collected and tested from three birds. T-tests were performed. * p < 0.05, ** p < 0.01, and ns for non-significant. The asterisks over columns represent a significant difference compared to the IBV infected group. Asterisks over lines represent a significant difference between mixed infection groups with and without bacteriophage treatments. Significantly lower viral titers were observed in the mixed infection group treated with bacteriophages.

Bacteriophage treatment significantly reduced APEC shedding after single APEC or mixed APEC and IBV challenge, with a gradual decrease of bacterial loads in lung tissues over time. In contrast, a non-treated and challenged group showed a significantly higher APEC load with a gradual increase over time especially at 9 and 15 dpc (Figure 5). Interestingly, bacteriophage treatment significantly reduced IBV shedding in the mixed infected group but not in the IBV alone infected group comparing to the mixed infected group without bacteriophage treatment. The bacteriophage treated group infected with IBV showed relatively comparable results to the infected non-treated group. Groups with single IBV infection and mixed APEC and IBV infection with bacteriophage treatment showed a reduction, but not statistically significant, of IBV comparing to single IBV infection without bacteriophage treatment, with the reduction only becoming statistically significant at 15 dpc (Figure 6).

## Discussion

Infectious bronchitis in particular complicated by mixed viral and/or bacterial infections is associated with catastrophic economic losses for the global poultry industry, and a significant threat for food security. Under field conditions complications caused by mixed IBV and APEC infections are linked with with accentuating respiratory signs in addition to airsacculitis, pericarditis and possible perihepatitis [28]. Under experimentally controlled conditions, mixed IBV and APEC infections can induce more pronounced clinical lesions in the air sacs that persist for a longer time [29]. In the present study samples were collected from birds with respiratory manifestations from a total of forty farms, and subjected to characterization based on currently circulating IBV and APEC strains. In addition, the efficacy of bacteriophage treatment in reducing APEC replication in the avian respiratory tract was studied *in vivo*, as well as their effect in reducing IBV pathogenicity after mixed APEC and IBV infections.

Out of forty farms, IBV was detected on 12 farms (30% of all farms examined). The high incidence of IBV in Egyptian poultry flocks suffering from respiratory manifestations (39–75.6%) has been recorded in several previous studies [8,10,30,31]. The range in the incidence rate is apparently due to the differences in the age of birds, with a higher incidence in 20–30 days old birds [8]. Based on partial spike protein gene sequencing, all positive IBV samples were clustered within the Egyptian variant IBV that belongs to the GI-23 genotype [18]. All recent molecular characterization of IBV circulating in Egypt revealed the circulation of two distinct genotypes, the GI-1 vicinal strains genotype and the GI-23 local variant genotype [32–34]. Despite wide spread vaccination with classical and/or variant strain based vaccines in Egypt, IBV outbreaks were frequently reported in vaccinated and non-vaccinated flocks of farms [16], indicating poor protection against the prevalent local variant strain [35].

In chickens, diseases associated with APEC cause significant economic losses due to mortality, decreased feed conversion rates, carcass contaminations and the high cost of its control and prevention. These losses in addition to the high prevalence of *E.coli* in poultry farms’ environment necessitate the use of antibiotics. However, the constant unwise use of antimicrobials in Egypt led to the high prevalence of multidrug-resistant strains that threaten human health and are associated with economic losses in the commercial poultry sector [36,37]. In the present study, 20% of the collected samples were positive for *E.coli*. 75% of them showed resistance to three or more antibiotics which were defined as multidrug resistant. This highlights the importance for the development of alternative therapeutic approaches preventing colibacillosis.

Bacteriophage therapy has been proven as an effective tool in the treatment of colibacillosis which initiated in the respiratory tract of chicken [38–40]. The ability to generate bacteriophages against selected bacterial species, serotypes or strains, that do not affect the commensal bacterial flora [41], make them safe in comparison to antibiotics and provide an alternative approach for eliminating pathogens.

In the present study, the efficacy of bacteriophage therapy in single and mixed infections with Egyptian IBV variant and/or APEC O78 has been evaluated. Results obtained revealed a significant higher persistence of APEC and IBV shedding together with higher morbidity and mortality in the mixed infection group comparing to single infected groups. This finding agrees with several previous studies [29,42,43]. Mechanisms behind the escalation of pathogenicity after mixed IBV and APEC infections are not yet fully understood. However, alteration of the immune response is most likely the cause not mechanical altering of the mucociliary barrier [29]. Bacteriophage treatment significantly reduced the severity of single APEC O78 challenge as well as a mixed infection with APEC and IBV. In brief, a high dose of bacteriophages administered intratracheally reduced the morbidity, prevented mortalities and significantly decreased APEC shedding compared to non-treated APEC challenge. Local application of bacteriophages at the site of APEC infection that is relatively inaccessible via the circulatory system, i.e. intra air sac administration, enables bacteriophages to eliminate the disease [44]. This explains the success of intrathoracic air sac injections in the reduction or prevention of mortality in several studies [38,44]. In the present study with a higher bacteriophage dose and intra-tracheal inoculation, APEC was significantly reduced in the bacteriophage treated lung tissue. In the case of mixed infections, bacteriophage treatment did not only reduce bacterial counts but also significantly reduced IBV detected in the lung, and associated with reduction in morbidity and mortality in the mixed infection group but not the single IBV infected group. These results show the efficient inhibition of APEC replication associated with a reduction of IBV severity during mixed infections.

In conclusion, the intra-tracheal administration of a high dose of bacteriophages limited APEC replication in the respiratory tract, and subsequently diminished the consequences of single and mixed infections with APEC and IBV. Bacteriophage treatment represents a promising tool for the prophylactic control of colibacillosis, and minimizes the consequences of mixed APEC and IBV infections.
